# Potential Linkage Between Zebra Mussel Establishment, Cyanobacterial Community Composition, and Microcystin Levels in United States Lakes

**DOI:** 10.3390/toxins17090447

**Published:** 2025-09-05

**Authors:** Feng Zhang, Jayun Kim, Ozeas S. Costa, Song Liang, Jiyoung Lee

**Affiliations:** 1 Environmental Science Graduate Program, The Ohio State University, Columbus, OH 43210, USA; 2 Division of Environmental Health Sciences, College of Public Health, The Ohio State University, Columbus, OH 43210, USA; kim.9582@osu.edu; 3 School of Earth Sciences, The Ohio State University at Mansfield, Mansfield, OH 44906, USA; costa.47@osu.edu; 4 Department of Environmental Health Sciences, School of Public Health and Health Sciences, University of Massachusetts, Amherst, MA 01003, USA; songliang@umass.edu; 5 Department of Food Science and Technology, The Ohio State University, Columbus, OH 43210, USA; 6 Infectious Diseases Institute, The Ohio State University, Columbus, OH 43210, USA

**Keywords:** microcystin, zebra mussel, cyanobacteria, phosphorus, nutrients

## Abstract

Zebra mussel invasion of North American lakes during the last century may play an important role in the occurrence of toxic cyanobacterial blooms. However, empirical evidence quantifying their influence on cyanobacterial community dynamics at broad spatial scales remains limited. Here, we analyzed data from the U.S. EPA National Lakes Assessment (>1000 lakes) to examine potential linkages among zebra mussels, cyanobacterial community composition, and cyanotoxin levels. The analysis results showed significant differences in cyanobacterial communities between lakes located in areas with and without established zebra mussel populations. The lakes with established zebra mussels exhibited significantly higher microcystin levels and cyanobacterial abundance, but lower phosphorus concentrations. Structural equation modeling was used to confirm and estimate the effect of zebra mussels on microcystin concentrations via different pathways. The results suggest three potential pathways whereby zebra mussels influence microcystin production: (1) altering phosphorus concentration; (2) increasing cyanobacterial abundance; and (3) shifting cyanobacteria community structure. The total effect of zebra mussel establishment resulted in an overall 1.40-fold net increase in microcystin level, which presumably resulted from three contributing factors: (1) a 1.06-fold increase through an increased cyanobacterial abundance; (2) a 1.53-fold increase through a selective force, resulting in increased cyanobacteria toxicity; and (3) a 0.86-fold decrease in microcystin level through total phosphorus decrease. The study highlights the potential role of zebra mussel invasion in altering cyanobacterial composition and influencing microcystin levels in U.S. lakes.

## 1. Introduction

One of the major consequences of lake eutrophication is the increase in blooms of cyanobacteria accompanied by a decrease in other groups of phytoplankton, namely chrysophytes, cryptophytes, chlorophytes and diatoms [1,2]. In addition to their adverse impact on ecosystem diversity, cyanobacterial blooms produce toxins that constitute a threat to both public and ecosystem health [3,4,5]. Cyanotoxins can poison humans and animals by drinking contaminated water or ingesting aquatic organisms that have bioaccumulated these toxins [6]. One group of commonly occurring cyanotoxins, microcystins, can cause acute death from liver damage and are potential carcinogens with long-term exposures [7,8]. Microcystins can be produced by toxic strains of numerous cyanobacterial species, such as *Microcystis*, *Nostoc*, *Oscillatoria*, *Dolichospermum*, *Planktothrix*, *Anabaenopsis* [9]. Among them, *Microcystis*, *Planktothrix*, and *Dolichospermum* are the most common [10,11].

Zebra mussels (*Dreissena polymorpha*) are invasive bivalves that have spread rapidly through the Laurentian Great Lakes watershed [12] and continue to spread throughout North American lakes [13]. Zebra mussels are extremely efficient at filter-feeding [14], leading to the filtering of suspended particles (including phytoplankton) and excreting nutrients, thereby physically altering the benthic habitat. By doing so, they change the composition and abundance of planktonic communities, and control aquatic ecosystem functions [15,16,17,18,19,20]. It has been suggested that this invasive bivalve has the potential to promote toxic blooms of cyanobacteria in the Great Lakes [21,22]. Dense summer blooms of toxic *Microcystis* were discovered three years after the initial colonization of zebra mussels in Lake Huron [23]. In Michigan lakes, zebra mussel presence has been associated with the dominance of *Microcystis aeruginosa* even in lakes with relatively low total phosphorus [24], a pattern contrary to the usual expectation that such blooms occur mainly in nutrient-rich waters. This association appears to arise from zebra mussel effects on grazing pressure and nutrient cycling, rather than from nutrient depletion by the blooms themselves.

Some studies have shown that *D. polymorpha* promotes an increase in cyanobacteria densities or their toxicity [25,26,27,28]; whereas other studies have shown that *D. polymorpha* decreases cyanobacteria densities [29,30] and a report shows high mortality of zebra mussel larvae under cyanobacterial bloom conditions [31]. A recent laboratory study also showed the bidirectional impact of *D. polymorpha* on phytoplankton community by nutrient excretion and selective grazing of phytoplankton [32]. Notwithstanding the information provided in these studies, the mechanisms for which zebra mussels affect cyanobacterial blooms in lakes are not fully investigated. Suggested mechanisms include: (1) selective filtration [22,33]; (2) nitrogen and phosphorus remineralization, which may support cyanobacterial blooms [34,35]; and (3) increased light penetration [36].

Although significant changes in phytoplankton community structure and composition have been identified with zebra mussel invasion [32,36], very few studies have focused on the effect of zebra mussels on cyanobacteria community structure and composition. To better understand the specific effects of zebra mussels on cyanobacterial community structure and microcystin levels in water, over one thousand lakes in the U.S. were investigated, using multivariate analysis of information obtained from the National Lakes Assessment (NLA) [37] and the U.S. Geological Survey Nonindigenous Aquatic Species (USGS-NAS) databases. Structural equation modeling was also used to explore potential pathways whereby zebra mussels could influence microcystin production.

## 2. Results

### 2.1. Cyanobacterial Community Structure in U.S. Lakes

**Figure 1:**
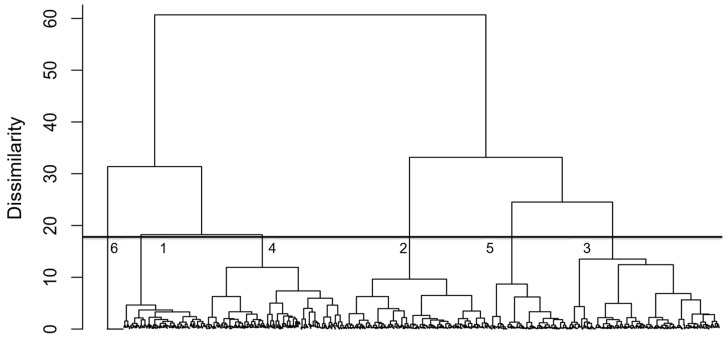
Agglomerative hierarchical clustering of cyanobacteria in U.S. lakes based on Ward’s linkage and Bray–Curtis distance, showing six clusters with distinct community assemblages. Numbers under the bar at a dissimilarity cutoff of around 18 indicate the cluster numbers. Cluster sizes were: Cluster 1 (138 lakes), Cluster 2 (244 lakes), Cluster 3 (252 lakes), Cluster 4 (231 lakes), Cluster 5 (132 lakes), and Cluster 6 (28 lakes, containing no cyanobacteria). SIMPER analysis showed *Microcystis* contributed most to between-cluster dissimilarity (9.67%), with an overall dissimilarity of 54.24%.

The NLA-2007 identified a total of 52 cyanobacteria genera, including *Dolichospermum*, *Microcystis*, and *Oscillatoria*, which are potential microcystin producers [10,11]. *Microcystis* and *Dolichospermum* were found in 599 (58.44%) and 593 (57.85%), respectively, of all lakes surveyed. The agglomerative hierarchical cluster analysis separated cyanobacterial communities into six clusters with distinct assemblages (Figure 1). We selected six clusters to avoid over-splitting the data: smaller dissimilarity thresholds produced too many small clades driven by low-level variation. Clusters 1–6 include 138, 244, 252, 231, 132 and 28 lakes, respectively. Cluster 1 was mainly composed of *Aphanocapsa* (12.22%), *Leptolyngbya* (12.16%), *Chroococcus* (11.91%), and *Dolichospermum* (10.32%); Cluster 2 contained 35.39% *Microcystis*, 20.83% *Oscillatoria*, and 12.44% *Dolichospermum*; Cluster 3 was predominantly *Microcystis* (64.70%); Cluster 4 was found to contain 19.77% *Dolichospermum*, 17.06% *Aphanizomenon*, and 14.65% *Aphanocapsa*; and Cluster 5 was composed of 61.11% *Microcystis* and 21.68% *Synechococcus* (Table 1). Cluster 6, consisting of 28 lakes (2.7% of the total), contained no cyanobacteria.

The similarity percentage (SIMPER) analysis identified the extent to which each genus contributed to the dissimilarity among clusters. *Microcystis* contributed most to the dissimilarity among clusters (9.67%), followed by *Dolichospermum* (8.09%), *Synechococcus* (7.13%), *Chroococcus* (6.62%), *Oscillatoria* (6.48%), *Aphanizomenon* (5.71%), and *Aphanocapsa* (5.49%). The overall dissimilarity among the six clusters was 54.24%.

**Figure 2:**
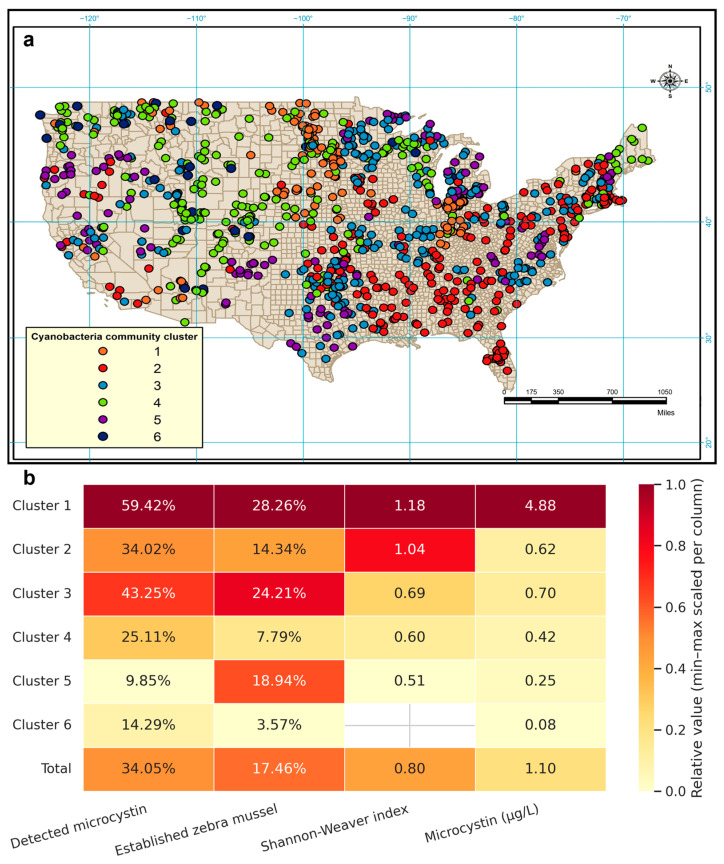
(**a**) Map showing the spatial distribution of six lake clusters identified by summer cyanobacterial community structure using agglomerative hierarchical cluster analysis of the National Lakes Assessment data. (**b**) Heatmap depicting characteristics of the six lake clusters, including the proportion of lakes with detected microcystin and established zebra mussel, the mean Shannon-Weaver index of cyanobacteria genera, and the mean microcystin concentration.

The distribution of the identified clusters shows a distinctive spatial clustering (Figure 2a). Permutational Multivariate Analysis of Variance (PERMANOVA) tests supports the results of the cluster analysis and differences between clusters are highly significant (*p* < 0.001). The Shannon-Weaver diversity index for each cluster also points to very distinctive cyanobacterial assemblages. In addition, the significantly different microcystin levels among clusters suggest a strong correlation between cyanobacterial community structure and microcystin level. The average Shannon-Weaver index of cyanobacteria genera was 0.80, ranging from 0 to 2.08. Lakes in Cluster 1 had the highest level of microcystin and also the highest cyanobacteria biodiversity, whereas lakes in Cluster 6 had the lowest level of microcystin and no cyanobacteria (Figure 2b). Spearman’s rank correlation between the Shannon-Weaver index of cyanobacteria genera and microcystin level was 0.221 (*p* < 0.001), indicating that higher cyanobacterial biodiversity was associated with higher microcystin levels.

### 2.2. Zebra Mussel Establishment and Cyanobacterial Community Structure

Nine 2-digit hydrological unit (HU) codes were recorded as having established zebra mussel populations; seven of these codes were repeatedly recorded in more than two different years. Regions frequently recorded with zebra mussel establishment included Mid Atlantic, Great Lakes, Ohio, Upper Mississippi, Lower Mississippi, Missouri, and Arkansas-White-Red regions (top-level 2-digit region HU codes). Among the 193 8-digit HU codes with zebra mussel establishment, only one location (drainage name: Lake Michigan) was recorded as “extirpated” (i.e., the population was previously established but subsequently disappeared without human removal, recorded in 2003). Most zebra mussel survey sampling occurred during the warm season: 80.2% of observations were collected between June and October. Chi-square test revealed that lake clusters are significantly associated with zebra mussel establishment (*p* < 0.001) (Figure 2b). Lakes in Cluster 1 had the highest probability (28.26%) of being in an area where zebra mussels were established while lakes in Cluster 6 had the lowest probability (3.57%). PERMANOVA showed a highly significant difference in cyanobacterial community structure between lakes located in areas that had been established with zebra mussels and those that had not (*p* = 0.001). SIMPER analysis demonstrated that the overall dissimilarity in cyanobacterial communities was 45.71% between the lakes located in areas with established zebra mussels and the lakes without them. SIMPER analysis also showed that *Microcystis* contributed most to the dissimilarity (8.29%), followed by *Dolichospermum* (8.08%), *Chroococcus* (7.03%), *Synechococcus* (6.79%), *Aphanizomenon* (5.79%), *Oscillatoria* (5.70%), and *Aphanocapsa* (5.48%).

### 2.3. Zebra Mussels and Cyanobacterial Blooms

**Figure 3:**
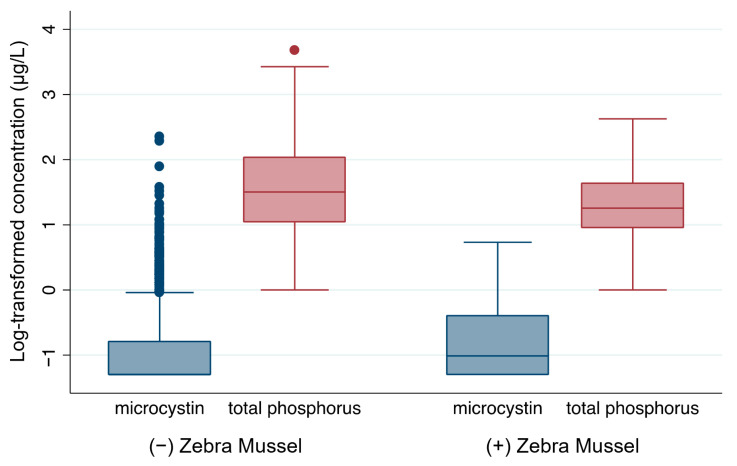
Box plots comparing microcystin and total phosphorus levels (log scale) between U.S. lakes with and without zebra mussel establishment. *N* = 848 and *n* = 180 for non-establishment and establishment, respectively. Dots indicate outliers, defined as values beyond 1.5× the interquartile range. Statistical comparisons between groups are reported in Table 2.

Among the lakes located in areas with established zebra mussels, 50.56% had detectable microcystin, while among lakes located in areas where zebra mussels had not been established, only 30.54% had detectable microcystin. Student’s t-test showed that microcystin levels (*t* = −2.73, *p* = 0.0065) and abundance of cyanobacteria (*t* = −2.51, *p* = 0.0127) were significantly higher in lakes located in areas where zebra mussels had been established (Table 2). Box plots of the log-transformed microcystin levels also indicated that lakes located in areas where zebra mussels had been established had higher microcystin levels (Figure 3). However, in lakes located in areas where zebra mussels were established, the total phosphorus level was significantly lower (*t* = 4.56, *p* < 0.0001), indicating that zebra mussels play an important role in nutrient budget. The nitrogen levels were not significantly (*p =* 0.4572) different based on status of zebra mussel establishment, indicating that the effect of zebra mussels on total nitrogen level was not significant. Zebra mussel establishment seemed to significantly increase the biodiversity of cyanobacteria genera (*t* = −2.87, *p* < 0.001).

### 2.4. Analysis of Potential Pathways Whereby Zebra Mussels Affect Microcystin Level

**Figure 4:**
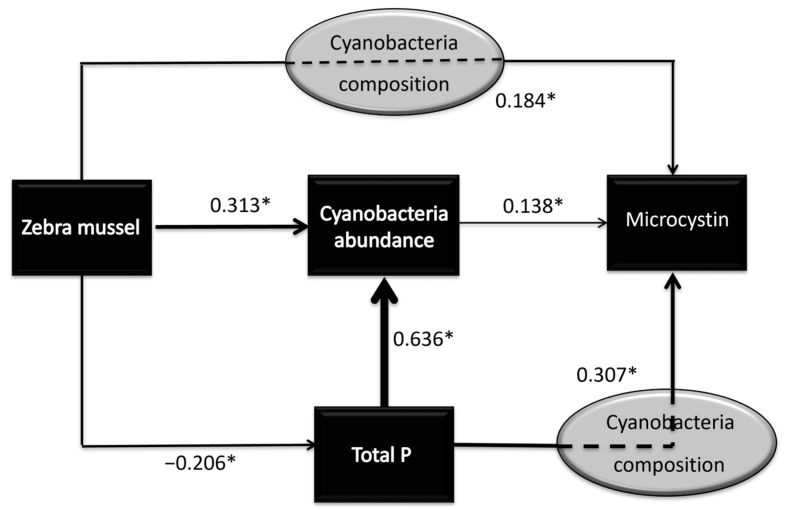
Structural equation models depicting the pathways for the influence of zebra mussel on microcystin level. Numbers on arrows represent standardized path coefficients, indicating the strength and direction of relationships between variables; positive values denote positive associations, negative values denote inverse associations. Asterisks (*) indicate highly significant paths at *p* < 0.001.

Zebra mussel establishment had significant relationships with total phosphorus level, cyanobacteria abundance, and microcystin level (Table 2). Based on the ability of zebra mussels to remove phosphorus and undertake selective filtration, we suspected that total phosphorus levels and cyanobacteria abundance were involved in the causal pathway of increasing microcystin levels in the presence of zebra mussels (Figure 4). Structural equation modeling confirmed the proposed causal pathway between zebra mussel establishment and microcystin level (Figure 4). In Figure 4, the numbers above the arrows indicate the strength of the direct relationship between the two variables in log terms. All the direct effects in the model were highly significant (*p* < 0.001). First, zebra mussel establishment led to a 1.61-fold decrease in total phosphorus level, which may lead to a decrease in cyanobacteria abundance and microcystin level. Second, zebra mussel establishment led to a direct 2.06-fold increase in cyanobacteria abundance, which may have contributed to increased microcystin levels. Third, zebra mussels have been shown to increase microcystin levels through an indirect effect by 1.53-fold (*p* < 0.001). This pathway could be the result of selective grazing in which toxic cyanobacteria are separated, mixed with mucus to form pseudofeces, and then expelled [38] thereby increasing the density of the toxic cyanobacteria. The net effect of zebra mussel establishment on microcystin level was a 1.40-fold increase (*p* = 0.005). This total effect consisted of a 0.86-fold decrease in microcystin level through reduced total phosphorus, a 1.06-fold increase through cyanobacteria abundance, and a 1.53-fold increase through other pathways, such as increase in toxic cyanobacteria.

## 3. Discussion

In the present study, we assessed the potential impact of zebra mussel establishment on summer cyanobacterial community structure and microcystin production using national-scale data from over 1000 lakes in the United States. Zebra mussel establishment was shown to affect cyanobacterial community structure, abundance of cyanobacteria, and microcystin level.

Cyanobacterial community structure was different in lakes located in areas with zebra mussel establishment as compared to those without. This may indicate that cyanobacterial community structure was influenced, possibly through selective grazing and nutrient removal by the zebra mussels. Lakes located in areas where zebra mussels were established had higher cyanobacteria abundance and biodiversity and lower phosphorus levels, which was consistent with phosphorus level observations in Saginaw Bay [39]. Nutrient levels play an important role in influencing cyanobacterial community composition [11,40]. Phytoplankton communities are sensitive to nutrient levels and the ratio of available nitrogen to phosphorus can play an important role in cyanobacterial community composition [11,41]. This could be a potential cause for differences in cyanobacteria community structure between lakes with and without zebra mussel establishment.

Grazing of zebra mussels on zooplankton could also lead to cyanobacteria community change, as zooplankton grazing and respiration are important drivers of the phytoplankton community [42]. Both nutrient cycling and selective grazing have been identified as mechanisms for the influence of zebra mussels on phytoplankton community structure and composition [27,37,43,44].

Both cyanobacterial community structure and cyanobacterial biodiversity were related to microcystin levels. Lakes with the highest cyanobacterial biodiversity also had the highest microcystin concentrations. A study on polar freshwater ecosystems also noted an association between cyanobacterial biodiversity and toxin production, suggesting a possible linkage between cyanobacterial biodiversity and microcystin levels [45]. Additionally, a study on Lake Erie found that cyanobacterial genetic diversity was associated with higher microcystin levels [46]. It should be noted that Shannon–Weaver index used to summarize cyanobacterial diversity can be biased when richness varies widely; therefore, future analyses could benefit from separating richness and evenness to confirm the observed patterns [47].

Microcystin levels were significantly higher in lakes located in areas where zebra mussels were established compared with lakes located in areas where they were not. A similar pattern was observed in 39 inland Michigan lakes where microcystin levels were higher in lakes with zebra mussels [26]. However, the mechanisms whereby zebra mussels influence microcystin levels are poorly understood. In the current study, higher cyanobacteria abundance and lower total phosphorus levels were observed in lakes located where zebra mussels were established. The reduction in total phosphorus after zebra mussel establishment has also been documented in the Great Lakes [16,48]. Previous studies have indicated that zebra mussels are extremely efficient in removing nutrients (N and P), especially phosphorus from lakes [49,50,51]. In our study, we only observed lower phosphorus levels in lakes located in zebra mussel invaded areas, which supports the assumption that zebra mussels effectively remove phosphorus from lakes [39]. Additionally, the reduction in phosphorus may reflect the complexity of phosphorus cycling (e.g., legacy P). A previous study in Lake Erie found that dreissenid presence altered the distribution of sedimentary phosphorus fractions [52]. Since total phosphorus and cyanobacteria abundance were both influenced by zebra mussel establishment, we believe them to be important factors involved in determining microcystin levels in lakes.

Structural equation modeling confirmed and quantified three possible ways whereby zebra mussel establishment could influence microcystin level (Figure 4). The assumption of a causal relationship is supported by our observed data and by the known the selective filtration ability of zebra mussels. Total phosphorus levels and cyanobacteria abundance were involved in the causal pathway of increasing microcystin levels in the presence of zebra mussels (Figure 4). By promoting cyanobacteria growth [53], and increasing cyanobacterial toxicity, high levels of phosphorus are usually directly implicated in increased microcystin levels [54,55]. Although nitrogen is an important factor in microcystin production, it was not proposed in a pathway due to the non-significant difference in nitrogen levels between lakes with and without zebra mussel establishment. Thus, the first pathway was by decreasing phosphorus concentration, which had a negative effect on microcystin production (Figure 4). The second was increasing cyanobacteria abundance, which had a positive effect on microcystin production. Third, zebra mussels may influence microcystin levels via other pathways, such as selective filtration/grazing and ejection of toxic cyanobacteria which may lead to the increased cyanobacteria abundance [56] and cyanobacterial toxicity [22]; both of which could increase microcystin production. Although the magnitudes of these reported significant pathways were moderate, the proportional changes associated with zebra mussel proliferation may have meaningful consequences for lake health and for human and wildlife exposure to microcystins.

In support of previously established data, we have demonstrated increased microcystin levels in lakes with zebra mussel establishment, showing that zebra mussel invasion led to increased toxic cyanobacterial blooms in inland lakes [22,26]. Zebra mussels have spread rapidly across the eastern half of North America after their establishment in the 1980s and their spread to the western U.S. was expected [57]. With the expansion of zebra mussels to other parts of the United States, we expect an increase in cyanobacterial blooms and consequently an increase in levels of microcystin, presenting a hazard to human and wildlife health and a detriment to recreational and other beneficial uses of inland lakes.

Some limitations of this study include that most lakes were sampled once for cyanobacterial measurements. Zebra mussel establishment status was assigned using HU codes from the USGS-NAS database; lakes located within invaded HU codes may not always show local evidence of invasion. By grouping lakes by HU code we may therefore underestimate zebra mussel effects, and the true influence on microcystin levels could be larger than reported. Furthermore, temperature, nitrogen, and nitrogen to phosphorus ratio should be examined in future work. Temperature can strongly influence cyanobacterial proliferation, particularly in seasonal studies; our current analysis is cross-sectional, and we infer that temperature effects are partially captured because cyanobacterial community composition trend (used in clustering) integrates temperature. Although previous studies emphasize the central role of phosphorus in bloom intensity and toxicity [11,58], nitrogen dynamics may also be relevant; future studies should explicitly evaluate changes in nitrogen and nitrogen to phosphorus ratio in relation to zebra mussel and cyanobacterial population changes in lakes. Despite these limitations, this study provides a nationwide cross-sectional trend that can guide future monitoring and experimental work and inform management and risk-assessment frameworks. Using more recent datasets (for example, newer NLA surveys) will be necessary to validate the potential linkages suggested here.

## 4. Conclusions

- Cyanobacterial community structure was different in lakes located in areas where zebra mussels were established compared with lakes located in areas where they were not;
- Cyanobacterial community structure and cyanobacterial biodiversity were positively correlated with microcystin levels;
- Microcystin levels and cyanobacteria abundance were significantly higher in lakes located in areas where zebra mussels were established compared with lakes located in areas where they were not;
- Total phosphorus levels were significantly lower in lakes located in areas where zebra mussels were established compared with lakes located in areas where they were not; possibly due to the nutrient removal ability of zebra mussels;
- There appeared to be a positive effect of zebra mussel establishment on microcystin occurrence, after controlling for total nitrogen and phosphorus levels. Alternatively, zebra mussel establishment can remove phosphorus and thereby suppress microcystin occurrence;
- Three possible ways in which zebra mussel establishment could influence microcystin levels were proposed and quantified. The first was a negative effect caused by phosphorus removal, which would reduce microcystin production whereas the second and third ways were assumed to be due to increase in cyanobacterial abundance and toxic cyanobacteria, which would result in a net positive effect on microcystin production.

## 5. Materials and Methods

### 5.1. Cyanobacteria Data

The NLA conducted by the U.S. EPA, Office of Water and Office of Research and Development, together with multiple organizations, provides a very comprehensive survey of lakes in the U.S. The NLA sampled 1028 lakes during the summer of 2007, representing the condition of about 50,000 lakes nationwide [37]. NLA measurements included indicators of water quality (nutrients, dissolved oxygen, and algal density); recreation (algal toxins and pathogens); physical habitat (lakeshore and shallow water habitat cover); and biology (phytoplankton and zooplankton, cyanobacterial abundance indicated by cells/mL, microcystin level, and trophic state indicated by nutrient level). At each lake site, crews collected samples at a single station located at the lake’s deepest point and at ten stations around the lake perimeter [37]. Single grab water samples were taken from just below the surface to a depth of up to 2 m to measure nutrients, chlorophyll-a, phytoplankton, and the algal toxin microcystin. Total microcystin was measured using ELISA, which has a detection limit of 0.1 μg/L. Microcystin concentrations below the detection limit were assumed to be half of the detection limit, 0.05 μg/L, which was already done by the U.S. EPA in the dataset. The sample for phytoplankton counting was fixed with Lugol’s iodine and identified to the lowest possible taxonomic level (usually genus) [59].

### 5.2. Zebra Mussel Data

Information on the distribution of zebra mussels was obtained from the USGS-NAS database (http://nas.er.usgs.gov/ (accessed on 22 August 2023)), a central repository of spatially referenced records of nonindigenous aquatic species. From this database, locations where zebra mussel had an “established” status—indicating evidence of successful reproduction (i.e., presence of multiple life stages or year classes) and overwinter survival—were identified. The zebra mussel data were combined with NLA data using the 8-digit HU code, which identifies a hydrological feature such as an area of a drainage basin. USGS has been using the HU code to track non-indigenous aquatic species for over ten years [60]. Since water bodies with the same HU code are hydraulically connected and geographically close, they are likely to share the same zebra mussel status, i.e., connected lakes are more likely to be invaded than non-connected lakes [61]. Zebra mussel establishment observed after 2007 was not considered while combining the two datasets.

### 5.3. Statistical Methods

To investigate differences in cyanobacterial community structure between lakes, we performed agglomerative hierarchical cluster analysis using the Bray–Curtis similarity coefficient [62]; a widely applied method in ecological studies. The cluster analysis was used to delineate groups of lakes with similar cyanobacterial communities. To stabilize the variance, raw abundance data were log(x + 1)-transformed and range standardized (subtracting the minimum and dividing by the range) prior to clustering. To minimize potential noise, low-prevalence genera in each lake (contributing ≤ 1% of that lake’s total abundance) were pooled into a single “Other” category before analysis. Clustering was conducted using Ward’s linkage, which has been shown to be the most robust method [63] to investigate differences in community structure. SIMPER procedure was used to identify those genera that contributed most to the difference between cyanobacterial communities in the groups. PERMANOVA was used to test whether there was a significant difference in cyanobacteria communities between lakes located in areas that have established zebra mussels and those that have not. To compare richness and evenness of cyanobacterial genera across the lakes, we used the Shannon–Weaver diversity index. This index, widely applied in aquatic microbial ecology for community comparisons, provides a balanced measure of diversity that is sensitive to changes in both dominant and rare taxa [64].

Student’s t-test was used to examine the effect of zebra mussel establishment on cyanobacteria-related parameters. Parameters deviating from normal distribution, judging from the normal Q-Q plot, were log-transformed before the t-test. Spearman correlation was used to identify factors that were correlated with the levels of microcystin. Structural equation modeling was used to estimate the effect of zebra mussels on microcystin concentration and identify pathways whereby zebra mussels influence microcystin concentration. We estimated the total effect, the effect through decreasing total phosphorus, and the effect through cyanobacterial abundance of zebra mussel on microcystin concentration. Before regression, microcystin levels, total nitrogen levels, and total phosphorus levels were log transformed, and cyanobacteria abundance was log + 1 transformed to meet assumptions of the model. Structural equation modeling is a powerful analysis technique widely used for causal inference [65]. It allows examination of a set of relationships between several variables and structural equation models are often visualized by path diagrams.

Agglomerative hierarchical cluster analysis and PERMANOVA were performed using R-Forge vegan package [66], a CRAN package for the analysis of ecological communities. The clustering tree was used to identify clusters that represent cyanobacterial community assemblages. The selection of the appropriate number of clusters depends on the purpose of the analysis and cluster interpretability [67]. Alternative selections in the number of clusters and cyanobacteria composition were used before making a final decision on the number of clusters. SIMPER calculations were conducted using PAST v. 2.17c [68]. Other statistical procedures were performed using SAS 9.3 (SAS Institute Inc., Cary, NC, USA).

## Figures and Tables

**Table 1:** Relative composition of cyanobacterial genera in the six identified lake clusters. Values are mean percentage of genus abundance. Only genera with a mean percentage of greater than 5% in at least one cluster are shown; genera below this threshold collectively account for the remaining percentages.

Genus	Cluster 1 (%) (*n* = 138)	Cluster 2 (%) (*n* = 244)	Cluster 3 (%) (*n* = 252)	Cluster 4 (%) (*n* = 231)	Cluster 5 (%) (*n* = 132)	Cluster 6 ^1^ (%) (*n* = 28)
*Dolichospermum*	10.32	12.44	7.30	19.77	1.54	0
*Coelosphaerium*	7.52	1.67	0.82	3.71	1.16	0
*Aphanizomenon*	6.71	0.88	5.61	17.06	1.32	0
*Aphanocapsa*	12.22	3.93	0.63	14.65	0	0
*Chroococcus*	11.91	5.29	1.14	7.04	0.77	0
*Leptolyngbya*	12.16	0.62	0.00	1.03	0	0
*Merismopedia*	5.77	4.22	1.22	5.43	1.04	0
*Microcystis*	6.48	35.49	64.70	2.59	61.11	0
*Oscillatoria*	0.06	20.83	1.74	0.91	3.08	0
*Phormidium*	6.73	0.08	0	3.74	0	0
*Synechococcus*	0.00	0.58	1.16	0.77	21.68	0

^1^ Cluster 6 contained no cyanobacteria.

**Table 2:** Relationship between zebra mussel establishment, cyanobacteria bloom-related parameters, and nutrient levels. Values represent the median for each variable. Variables showing statistically significant differences (*p* < 0.05) between conditions with and without zebra mussel establishment are shown in bold.

	Zebra Mussel Status of the Area		Student’s *T*-Test	
Variable	Zebra Mussel Not Established (*n* = 848)	Zebra Mussel Established (*n* = 180)	*T*-Statistic	*p*-Value
**Microcystin** (μg/L)	0.05	0.10	−2.73	0.0065
Nitrogen (μg/L)	576	627	−0.74	0.4572
**Phosphorus** (μg/L)	32	18	4.56	<0.0001
**Cyanobacteria** (cells/mL)	5248	6157	−2.51	0.0127
*Microcystis* (cells/mL)	239	508	−1.21	0.2250
***Dolichospermum*** (cells/mL)	10	97	−5.64	<0.0001
Chlorophyll-a (μg/L)	8.67	6.40	0.85	0.3960
**Cyanobacteria biodiversity**	0.74	1.03	−2.87	<0.0001

## Data Availability

The data used in this study is publicly available at the following link, https://www.epa.gov/national-aquatic-resource-surveys/nla (accessed on 3 June 2023).

## Abbreviations

The following abbreviations are used in this manuscript:

NLA	National Lakes Assessment
SIMPER	similarity percentage analysis
PERMANOVA	Permutational Multivariate Analysis of Variance
HU	hydrologic unit
